# Has the Time Come to Stop Using the “Standardised Mean Difference”?

**DOI:** 10.32872/cpe.6835

**Published:** 2021-09-30

**Authors:** Pim Cuijpers

**Affiliations:** 1 Department of Clinical, Neuro and Developmental Psychology, Amsterdam Public Health research institute, Vrije Universiteit Amsterdam, Amsterdam, The Netherlands; Philipps-University of Marburg, Marburg, Germany

**Keywords:** effect size, standardised mean difference, meta-analysis, outcome studies

## Abstract

**Background:**

Most meta-analyses use the ‘standardised mean difference’ (effect size) to summarise the outcomes of studies. However, the effect size has important limitations that need to be considered.

**Method:**

After a brief explanation of the standardized mean difference, limitations are discussed and possible solutions in the context of meta-analyses are suggested.

**Results:**

When using the effect size, three major limitations have to be considered. First, the effect size is still a statistical concept and small effect sizes may have considerable clinical meaning while large effect sizes may not. Second, specific assumptions of the effect size may not be correct. Third, and most importantly, it is very difficult to explain what the meaning of the effect size is to non-researchers. As possible solutions, the use of the ‘binomial effect size display’ and the number-needed-to-treat are discussed. Furthermore, I suggest the use of binary outcomes, which are often easier to understand. However, it is not clear what the best binary outcome is for continuous outcomes.

**Conclusion:**

The effect size is still useful, as long as the limitations are understood and also binary outcomes are given.

It was a historical event for the field of clinical psychology. In his presidential address to the American Educational Research Association in 1976 in San Francisco, Gene Glass not only coined the term “meta-analysis” but he also introduced the basic ideas of modern meta-analyses (Hunt, 1997). This event is broadly considered as the starting point of modern meta-analyses (Hunt, 1997). Since then this method has conquered the field of clinical psychology and beyond, and meta-analyses have become the standard for integrating the results of multiple studies on the same research question into one estimate of the effects or associations. Meta-analyses are now considered to be the gold standard for estimating the effects of interventions and are at the basis of treatment guidelines for mental health and other problems as well as policy recommendations about treatments.

Glass brought forward two basic ideas that are at the core of modern meta-analyses. The first idea he brought forward was the ‘standardized mean difference’, or what is often called the ‘effect size’. The effect size indicates the difference between two conditions after the intervention in terms of standard deviations instead of actual scores on an outcome instrument. This makes the outcomes ‘standardised’ and therefore they can be compared across studies. The other basic idea of meta-analyses that Glass brought forward was that these standardised outcomes can be pooled across studies, while weighting them based on the size of the samples. This pooling of the standardised outcomes results in one overall estimate of the true effect size across multiple studies.

It is now 45 years ago that these two basic ideas were introduced. The second idea, the pooling of outcomes according to the size of the study, has hardly been disputed since the introduction by Glass. But the idea of the standardised mean difference has been more controversial over the years. In this paper, I will focus on the standardised mean difference. I will discuss whether this is still the best way of indicating the outcomes of interventions or associations between variables or whether it is better to start using binary outcomes instead. I will call the standardised mean difference the ‘effect size’ which is in fact not correct (Higgins & Green, 2011), but I will still do that to increase the readability of this paper.

## The Effect Size

It was a brilliant idea to indicate the difference between two groups in terms of the standard deviation of the outcome measure, instead of the actual difference in scores between the groups. This not only allows to compare these outcomes across different studies regardless of the outcome instrument used, but it also gives an indication of the size of the effect. Previous research often only indicated whether the difference between two groups was significant or not. However, that is not very informative and does not say anything about the size of the difference. Whether or not a difference is significant depends on the size of the sample, and even a tiny difference becomes significant when the sample size is large enough. The effect size solved this problem, because it goes beyond significance levels and indicates how large the difference is. Cohen suggested that an effect size of 0.2 should be considered as small, 0.5 as moderate and 0.8 as large (Cohen, 1988).

However, the use of effect sizes also has several important limitations. One important limitation is that it is still a statistical concept. It may indicate the strength of an outcome, but it still cannot say anything about the clinical relevance of the outcome (Cuijpers, Turner, Koole, van Dijke, & Smit, 2014). The clinical relevance of an effect size depends on the content. For example, an effect size of 0.1 would be considered a major breakthrough if years to mortality would be the outcome. And effect size of 0.1 with “knowledge of depression” as outcome, however, would be considered trivial by most people. This means that the categories of small, moderate and large effect sizes, as given by Cohen (1988) may be misleading because the effect size depends too much on what the outcome actually is. It should be noted that this was fully acknowledged by Cohen.

One solution to the problem that the effect size is a statistical concept, could be the use of the ‘Minimal clinically important difference’ (MCID; McGlothlin & Lewis, 2014). The MCID is the smallest difference in score considered clinically worthwhile by the patient and it captures both the magnitude of improvement and the value the patient places on that improvement. For example, it was found in one study that a reduction of 17.5% from baseline to post-test on the BDI-II can be considered as the Minimal clinically important difference (Button et al., 2015). Currently, it is also possible to convert different measures into one common metric (e.g., Wahl et al., 2014), making the use of the effect size no longer needed.

The effect size has other problems. For example, it assumes that different outcome scales are linear transformation of each other and the standard deviation units are indeed the same across all studies (Cummings, 2011). These assumptions do not necessarily need to be true in all situations. Furthermore, the effect size may be influenced by how narrow the inclusion criteria are (Cummings, 2011). If a trial only includes participants with a narrow severity range at baseline, it can be expected that the distribution of the severity at post-test is also relatively narrow. If patients with a broader severity range are included, the distribution of severity will be broader as well. This implies that if two trials, one with a narrow severity range and one with a broad severity range, show the same absolute difference (points on a severity scale), the effect size can still vary widely, because the distribution differs across the two studies.

## What Does the Effect Size Mean?

The most important problem of the effect size is, however, that it is so difficult to explain what it exactly means to non-scientists. Imagine a patient who considers to accept a treatment and asks the clinician what the chances are to get better after treatment. The clinician will have to say something like “if you get the treatment you will score 0.5 standard deviation lower on the outcome measure than not receiving the intervention”. Of course a patient has no clue for what this actually means, and many clinicians also find it hard to understand what it means.

There are some solutions to this problem. One older solution is to transform the effect size into the ‘binomial effect size display’ (BESD) (Rosenthal & Rubin, 1982). The BESD reduces an outcome to a simple dichotomy (for example whether a score is below or above the mean on the outcome instrument) and indicates the difference between the two treatment groups (e.g., therapy and control) in percentages of participants who score below (or above) the mean (Randolph & Edmondson, 2005). For example, an effect size of 0.2 indicates a difference of 0.10 in the proportion of participants reaching this threshold. One could say that such a value of the BESD means that 45% of the control group and 55% of the treatment group had reached the threshold of 'success'. However, this is still a relative outcome and can in no way be interpreted as if 55% of the participants will score below the mean of the outcome measure.

Another way to make the effect size easier to interpret is to transform it into the number-needed-to-treat (NNT). The NNT indicates the number of patients that have to be treated in order to have one more positive outcome than no treatment (or an alternative treatment) (Laupacis, Sackett, & Roberts, 1988). There are several ways to transform the effect size into the NNT (da Costa et al., 2012; Furukawa & Leucht, 2011), but all are based on the normal distribution of the outcome measure and a cut-off on this normal distribution for a ‘positive outcome’. However, again it is not clear what this ‘positive outcome’ exactly is and the NNT still does not answer the question of the patient what the chances are to get better after treatment. Transforming the effect size into the NNT is, however, done by many meta-analyses to make the outcomes easier to interpret from a clinical point of view.

## Moving to Binary Outcomes?

Binary outcomes are easier to understand than effect sizes. For example, in a trial the researchers can calculate the proportion of participants that respond (for example defined as a 50% reduction in symptoms from baseline to post-test) in the treatment and control group. They can also calculate the proportion of participants who recover completely (for example by scoring below a cut-off on a symptom measure), who reliably improved, or who reliably deteriorated, or dropped out from treatment. These binary outcomes can answer the question of the imaginary patient that we presented earlier very well. The patient will hear an exact chance of getting better after the treatment compared to no treatment.

For example, we recently conducted a meta-analysis of psychotherapies for depression (Cuijpers, Karyotaki, de Wit, & Ebert, 2020) and found that the effect size for psychotherapy versus control conditions was *g* = 0.72, 95% CI [0.67, -0.78]. That is a considerable effect according to the criteria of Cohen. But what does it really mean? What is the chance of getting better for a patient receiving therapy compared to the chance in the control conditions? In another recent meta-analysis, we calculated the exact proportions of response (50% reduction of symptoms between baseline and post-test) (Cuijpers, Karyotaki, Ciharova, Miguel, Noma, & Furukawa, 2021) for psychotherapies with at least 10 trials for which the response rate was reported or could be estimated using a validated method (Furukawa, Cipriani, Barbui, Brambilla, & Watanabe, 2005). We found that the response rate for psychotherapies was 41% (using the most conservative estimate), while the response rate was 17% in the usual care groups. This is definitely more informative for patients and clinicians than the effect size. It shows for example that about 60% of patients do not respond after therapy and that the proportion of patients responding to usual care is really very low. The effect size gives no indication at all for such outcomes. It just says that the effect are “large”, but this “hides” in a way that the majority of patients still don’t respond to treatment.

## Disadvantages of Binary Outcomes

So does this solve the problem? Should we all move away from the effect size and instead use binary outcomes? Unfortunately, binary outcomes also have problems. Maybe the most important problem is that outcomes may be best considered as a continuous phenomenon and not as a binary outcome. One can use binary outcomes that are informative, such as response or remission, but that does not solve the problem that in principle outcomes are still continuous. Another problem is that in individual trials binary outcomes have less statistical power to find significant differences between treatment and comparison conditions. Furthermore, there is no way to decide what the best binary outcome is. In many trials on psychological treatments the Reliable Change Index (RCI) is used (Jacobson & Truax, 1991), a psychometric criterion used to evaluate whether the change between baseline and post-test is considered statistically significant (the difference between baseline and post-test means divided by the standard error of the difference between the two scores is greater than 1.96, conservatively assuming a Cronbach’s alpha of 0.75) (Jacobson & Truax, 1991). Other studies use the response (50% reduction in symptoms from baseline to post-test) or remission (scoring below a cut-off on a rating scale indicating the return to ‘normal’ functioning) as the main outcome. There is no way to decide what the most important binary outcome is and that may therefore vary widely across studies, making meta-analyses of these outcomes more complicated. But it also makes the answer to the question of the patient more complicated. It can be said what the chance of getting better is, but what getting better actually is, is not so clear.

Another problem with reporting the chance of getting better in the treatment and control conditions is that these chances can be very well reported for individual trials, but pooling them in meta-analyses may be problematic. The problem with exact percentages is that when you pool them, the heterogeneity of the outcome is often very high. Heterogeneity indicates the variability in the outcomes of the included studies in a meta-analysis. If heterogeneity is too high that means that the outcomes are too different from each other to allow pooling. And that is typically the case when proportions are pooled. But on the other hand, these outcomes are so important for patients and clinicians, that one could make the case to pool anyway, but always say that the outcomes can vary considerably.

Usually, binary outcomes in meta-analyses are not reported in terms of absolute percentages, because of the high heterogeneity. In most cases binary outcomes are reported in terms of relative outcomes, such as the Relative Risk (*RR*) or the Odds Ratio (*OR*). The *OR* indicates the odds of getting better in the treatment group compared to the control group. This is also difficult to interpret, because it is not immediately clear what the odds are and it can be argued that the *OR* should be avoided as well because it is not clear what it means (Higgins & Green, 2011). The *RR* is easier to interpret. An *RR* of 1.40 for example indicates that the chance of getting better is 40% higher in the treatment group than in the control group. Sometimes the NNT is also used. The NNT is actually the inverse of the Risk Difference (*RD*). So if 60% get better in the treatment group and only 40% in the control group, the *RD* is 20% and the NNT is 5 (1/0.20).

But all relative outcomes do not answer the question of the patients what the chances are of getting better after the treatment. In order to answer that, it cannot be avoided to give the actual chances.

## Conclusion

So should we stop using the effect size and instead move to reporting the proportions of participants who improve in the treatment and the control group? I don’t think that is needed. Many studies already give the effect size and one or more binary outcomes. That is probably the best solution.

But we should avoid to obscure outcomes by just saying that a treatment is effective and the effect size is large, moderate or small. Such a statement can mean many different things. A large effect size can still indicate that many people don’t get better, and a small effect size can be a major breakthrough. It is important to add in trials but also in meta-analyses what the effect sizes exactly mean in terms of relative but also absolute binary outcomes.
